# Effectiveness and safety of statins on outcomes in patients with HIV infection: a systematic review and meta-analysis

**DOI:** 10.1038/s41598-022-23102-2

**Published:** 2022-10-27

**Authors:** Njeodo Njongang Vigny, Kwadwo Osei Bonsu, Amudha Kadirvelu

**Affiliations:** 1 Department of Medical Laboratory Science, Faculty of Health Sciences, University of Buea, Buea, Cameroon; 2 Department of Medical Laboratory Science, School of Engineering and Applied Sciences, Institut Universitaire de La Côte, Douala, Cameroon; 3 School of Pharmacy, Memorial University of Newfoundland and Labrador, St. John’s, NL Canada; 4 Jeffrey Cheah School of Medicine and Health Sciences, Monash University Malaysia, Jalan Lagoon Selatan, Bandar Sunway, 47500 Subang Jaya, Selangor Malaysia

**Keywords:** Biomarkers, Cardiology, Diseases, Molecular medicine

## Abstract

Statins are hypolipidaemic in human immunodeficiency virus (HIV) positive individuals. However, their effect on all-cause mortality and rate of discontinuation is unclear. We conducted a systematic review to evaluate the impact of statins on all-cause mortality, discontinuation rates, and risk of adverse effects among HIV patients on highly active antiretroviral therapy (HAART). We searched four electronic databases from inception until October 2021 for trials and cohort studies evaluating the effects of statin treatment versus placebo in HIV patients. Forty-seven studies involving 91,594 patients were included. Statins were associated with significantly lower risk of discontinuation (RR, 0.701; 95% CI 0.508–0.967; *p* = 0.031). The risk of all-cause mortality (RR, 0.994; 95% CI 0.561–1.588; *p* = 0.827), any adverse effects (RR, 0.780; 95% CI 0.564–1.077; *p* = 0.131) and, diabetes mellitus (RR, 0.272; 95% CI 0.031–2.393; *p* = 0.241) with statin treatment were lower but not statistically significant compared to placebo/control. Statin treatment was associated with a trend of higher but statistically insignificant risk of myalgia (RR, 1.341; 95% CI 0.770–2.333; *p* = 0.299), elevated creatine kinase (RR, 1.101; 95% CI 0.457–2.651; *p* = 0.830) and liver enzyme activities (RR, 1.709; 95% CI 0.605–4.831; *p* = 0.312). Clinicians should consider the nocebo effect in the effective management of PLWH on statins, who present with common adverse effects such as myalgia and, elevated levels of creatine kinase and liver enzymes.

## Introduction

Globally, human immunodeficiency virus (HIV) infection still poses a public health threat. While the burden of HIV has steadily risen over the last decade^1^, acquired immunodeficiency deficiency syndrome (AIDS)-related mortality has dropped as a result of the rapid uptake of highly active anti-retroviral therapy (HAART) over the past decade. Moreover, there is a substantial and growing burden of cardiovascular disease (CVD) among people living with HIV (PLWH), which is driven by an interaction between classical and HIV-specific factors, as well as HAART-related dyslipidaemia^2^.

In PLWH, statins have a hypolipidaemic effect^3^. However, trials involving PLWH and statins have not indicated a significant reduction in mortality^4–6^. In addition, a prospective cohort research and two randomized controlled trials (RCTs) have demonstrated that adverse effects such as myalgia, elevated transaminase and creatine kinase (CK) levels, nausea, and diarrhoea are more prevalent in statin-exposed PLWH on HAART compared with those on placebo^7–9^. Data about rate of discontinuation with statins in PLWH has been conflicting. Two randomized controlled trials and one cohort study found greater discontinuation rates among statin users, whereas two randomized trials found higher discontinuation rates among placebo users^7–11^.

Recent cohort studies involving PLWH did not demonstrate a statistically significant reduction in mortality between statin users and non-users^4–6^. Nonetheless, substantial limitations were observed in these cohorts; first, only 8% of PLWH were taking statin, second, the number of PLWH without statins was three times that of statin users, and third, the mortality rate was too low (8.3%) to identify a statistically significant difference.

Now, there is paucity of information about the effectiveness and safety of statin therapy for the prevention of clinical outcomes in PLWH. Therefore, we conducted a systematic review and meta-analysis to evaluate the efficacy and safety of statin therapy with respect to the risk of adverse events, discontinuation, and mortality in comparison to control/placebo in PLWH.

## Methods

The PRISMA (Preferred Reporting Items for Systematic Reviews and Meta-Analyses) and more recent PRISMA-S guidelines were followed in the current meta-analysis^12,13^. No approval from an institution-related review committee was obtained for our study registered at Open Science Framework (https://doi.org/10.17605/OSF.IO/P5ETQ).

### Search strategy

Relevant articles in MEDLINE (Ovid), EMBASE (Ovid), GLOBAL HEALTH (CABI), and Cochrane Library were explored from inception to October 2021 irrespective of language using the terms: ((statin) OR (HMG-CoA reductase inhibitors) OR (atorvastatin) OR (rosuvastatin)OR (fluvastatin) OR (lovastatin) OR (pitavastatin) OR (simvastatin)) AND (HIV OR AIDS OR (human AND immunodeficiency AND virus)).

### Study selection

All titles and abstracts from databases search were exported to Rayyan software and identical records were deleted. The remaining records were screened by two co-authors (NNV and KOB) and any dissimilarities were resolved by consensus. Full-text articles were assessed and included in the meta-analysis if they met all the following criteria: (i) human studies, (ii) PLWH with/without HAART aged ≥ 18 years, (iii) statin treatment for primary or secondary prevention of cardiovascular event or mortality respectively, and (iv) ≥ 3-week follow up. In contrast, studies in a non-human setting and duplicate reporting were excluded.

### Data extraction

Two authors (NNV and KOB) independently extracted information from each eligible study using a standardized data extraction form. We extracted data on: author’s name; year of publication; sample size; study design; location of study; duration of follow-up; population and setting; baseline characteristics; type, duration and dosage of statin use; mortality from cardiovascular disease and any cause; discontinuation rates; adverse effects; and age and sex of study participants. Discrepancies in extractions were resolved by discussion. The primary endpoint was all-cause mortality. Discontinuation (owing to self-discontinuation of statin, loss to follow-up, or statin toxicity) and incidence of any adverse effects (i.e., myalgia, elevated CK and liver enzyme levels, and gastrointestinal events) and diabetes mellitus with statin treatment were secondary endpoints.

### Quality and validity assessment

Two review authors (NNV and KOB) independently rated the quality of included RCTs as low, some concerns, or high risk of bias with the Cochrane Collaboration Risk of Bias tool version two (CCRBT)^14^ and cohort studies were rated as good, fair, or poor using the Newcastle–Ottawa Scale^15^.

### Data synthesis and analysis

Median (± interquartile range) or mean (± standard deviation) was used to present continuous variables. Lipid profile values were reported in mmol/L [1 mmol/L (TC/HDL/LDL) = 38.67 mg/dL; 1 mmol/L (TG) = 88.57 mg/dL]. The risk ratios (RR) and 95% confidence intervals (CI) for all outcomes were computed using random-effects model. Statistical heterogeneity and homogeneity were estimated using I^2^ statistic and Cochran Q test respectively. I^2^ values of 25%, 50%, and 75% represent mild, moderate, and extensive statistical inconsistency respectively. We performed meta-regressions and subgroup analyses to determine sources of clinical heterogeneity across studies. Our subgroup analyses explored the impact of the study type (RCT, cohort studies), statin type, statin class and statin dosing intensity on treatment outcomes where applicable. The ACC/AHA classification of intensity of statin dosing was used to characterize intensity of statin dosing in the included studies^16^. Funnel plots and Begg adjusted-rank correlation and Egger regression asymmetry tests^17,18^ were used to assess publication bias. To test the robustness of our results, we conducted several sensitivity analyses. All analyses were performed using Comprehensive Meta-analysis Software version 3.0 (Biostat, Englewood, NJ, USA) and R Statistical Software version 4.1.2 (R Foundation for Statistical Computing, Vienna, Austria, URL http://www.R-project.org, 2020).

## Results

### Study selection

**Figure 1:**
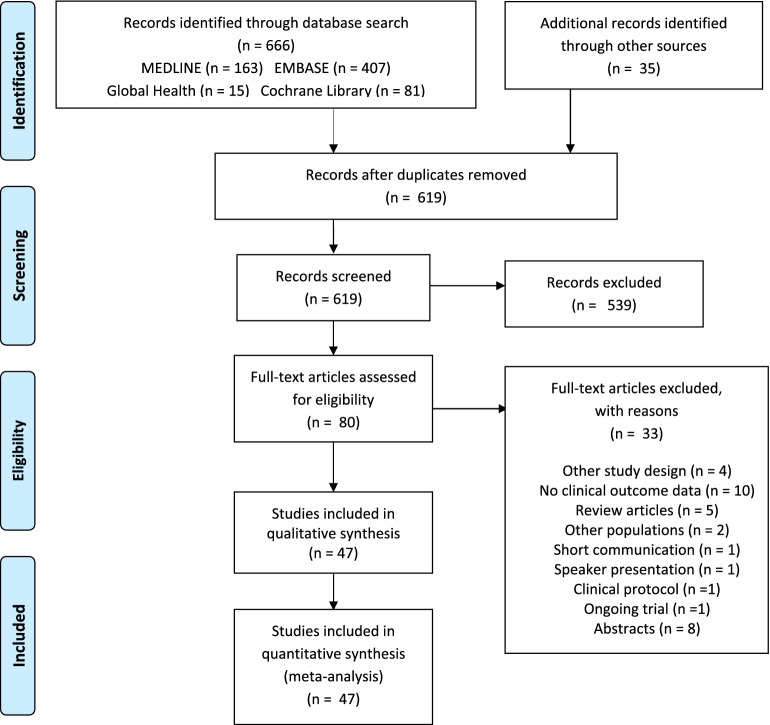
PRISMA flow diagram for the systematic review study selection.

Our systematic literature search identified 666 articles; titles and abstracts were screened for the remaining 619 articles after removal of duplicates (Fig. 1). After full-text assessment of 80 articles, 33 studies were excluded. 47 articles met the inclusion criteria and were published from 2001–2021. Ten (21.3%) studies enrolled at least 1,000 (1,182–40,029) participants and utilized rosuvastatin 10–20 mg^8,11,19–26^. Pravastatin 40 mg^20,27–35^ were evaluated in 10 studies while, 4, 3, 2 and 2 other studies utilized atorvastatin 10–40 mg^9,10,20,36^, pitavastatin 2–4 mg^34,37,38^, simvastatin 10–20 mg^36,39^, and fluvastatin 20–40 mg^40,41^ respectively (Fig. 1).

### Study characteristics

Participant characteristics (Table 1) of the included studies have been summarized (see Supplementary Table S1 online) and twenty-one RCTs involving 1,482 PLWH on HAART (880 statin users vs. 602 statin non-users) were included. Majority of the studies were conducted in North America (USA = 24 and Canada = 2) while the rest in Europe (Italy = 7, France = 3, UK = 2, Germany = 1, and Denmark = 1), Asia (Thailand = 2 and China = 1), Australia (n = 3), and Uganda (n = 1). 17.0% (91,594) of females were enrolled in the 47 studies, with a median age of 46.5 years [min–max (18.0, 56.8)]. At baseline, median CD4 + T-cell count was 493 × 10^6^ cells/L [IQR (85, 682)], mean time from HIV diagnosis was 11.5 years [IQR (0.98, 16.0)], and average HAART duration was 45.0 months [IQR (3.0, 147.0)]. The follow-up duration varied from 3.0–197 weeks (median = 12.0 weeks). Five cohort studies evaluated all-cause mortality. Fourteen RCTs and one cohort assessed discontinuation rates while twelve RCTs and one cohort reported adverse events.

**Table 1:** Baseline characteristics of enrolled patients.

	Median	Interquartile range
Age (years, min–max)	46.5	18.0–56.8
Gender Female	17.0	0–80.8
Body mass index	25.1	22.4–30.0
Caucasian ethnicity	65.5	22.5–100.0
Men having sex with men	43.8	18.2–70.0
Bisexuals	34.9	12.0–71.1
Intravenous drug user (previous or present)	13.2	0.0–45.7
Hypertension	25.9	6.7–66.0
Smoke	41.6	15.7–76.0
Diabetes Mellitus	12.0	3.8–40.0
HBV infection	6.5	3.0–36.0
HCV infection	14.6	4.7–34.9
Length of follow-up (weeks)	12.0	3–197.0
Total cholesterol (mmol/L)	5.5	3.2–7.4
HDL (mmol/L)	1.2	0.9–1.7
LDL (mmol/L)	3.4	2.4–4.3
Triglycerides (mmol/L)	2.7	1.3–9.4

### Effect of statin treatment on all-cause mortality in HIV-infected patients

**Figure 2:**
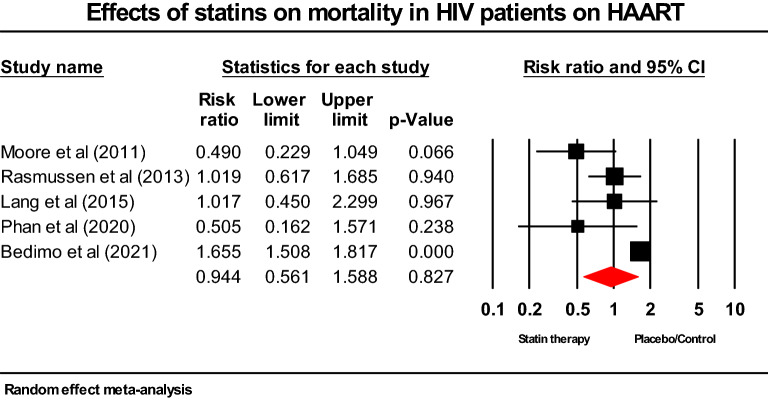
Forest plot comparing the impact of statins versus placebo on all-cause mortality in HIV-infected patients on HAART.

Statins were comparable to placebo/control in reducing all-cause mortality in PLWH with/without HAART (RR; 0.994; 95% CI 0.561–1.588; *p* = 0.827) (Fig. 2).

### Effect of statins on discontinuation rates among patients with HIV

**Figure 3:**
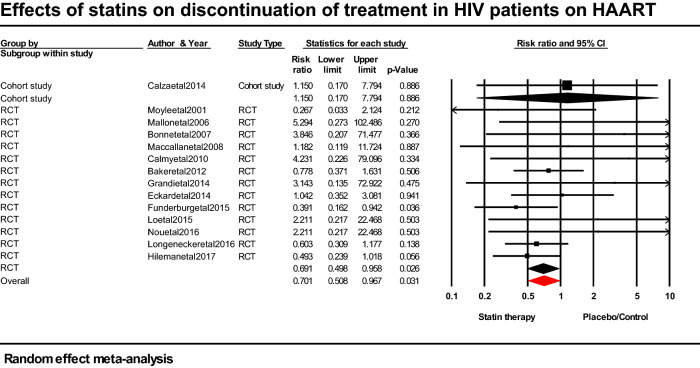
Forest plot comparing the impact of statins versus placebo on discontinuation of treatment in HIV-infected patients on HAART.

Statins in HAART exposed or unexposed PLWH is associated with lower risk of discontinuation (RR; 0.701; 95% CI 0.508–0.967; *p* = 0.031) compared with placebo/control in these patients–control group (Fig. 3).

### Effect of statin treatment on incidence of adverse events in HIV-infected patients

**Figure 4:**
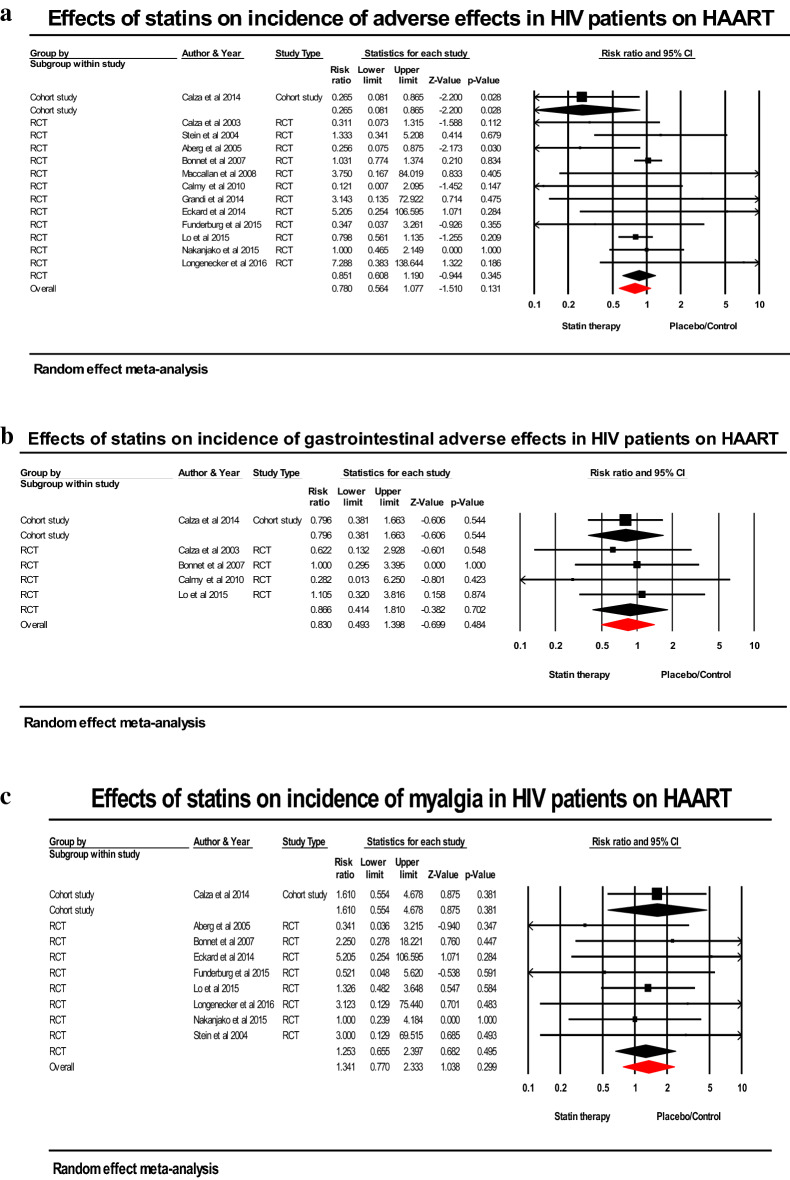

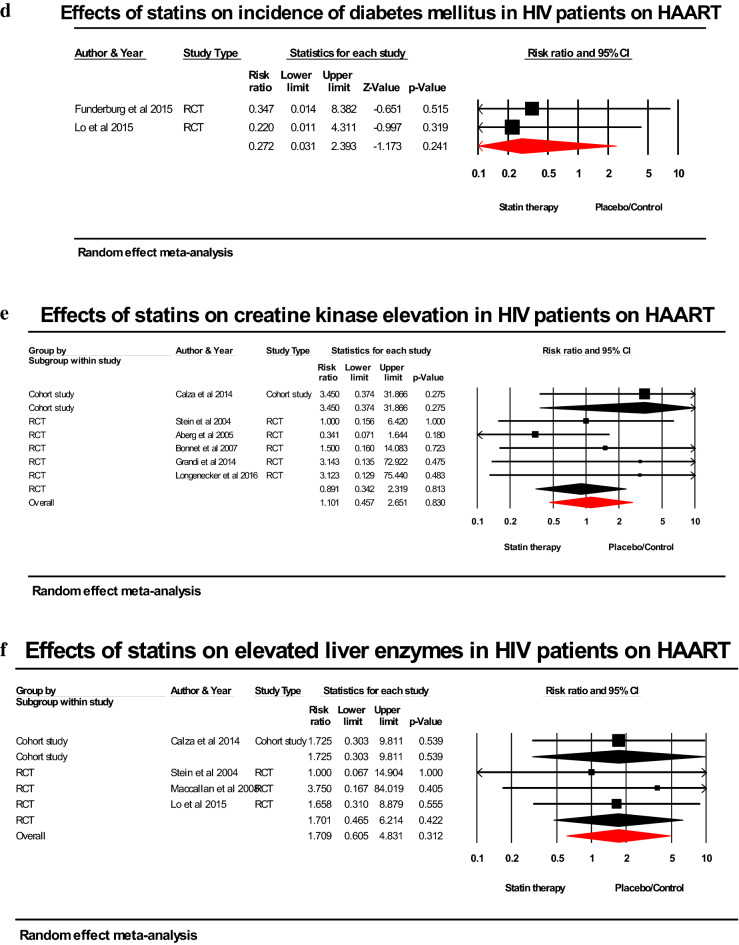
(**a**) Forest plot comparing the impact of statins versus placebo on incidence of any adverse effects in HIV-infected patients on HAART. (**b**) Forest plot comparing the impact of statins versus placebo on incidence of gastrointestinal adverse effects in HIV-infected patients on HAART. (**C**) Forest plot comparing the impact of statins versus placebo on incidence of malgia in HIV-infected patients on HAART. (**d**) Forest plot comparing the impact of statins versus placebo on incidence of diabetes mellitus in HIV-infected patients on HAART. (**e**) Forest plot comparing the impact of statins versus placebo on incidence of creatine kinase elevation in HIV-infected patients on HAART. (**f**) Forest plot comparing the impact of statins versus placebo on incidence of elevated liver enzymes in HIV-infected patients on HAART.

Statin therapy was not significantly associated with lower incidence of any adverse effects (RR; 0.780; 95% CI 0.564–1.077; *p* = 0.131), gastrointestinal adverse effects (RR; 0.830; 95% CI 0.493–1.398; *p* = 0.484), and diabetes mellitus (RR; 0.272; 95% CI 0.031–2.393; *p* = 0.241) compared to placebo/control. There was a non-significant increase in the risk of myalgia (RR; 1.341; 95% CI; 0.770–2.333; *p* = 0.299) and elevated liver enzymes (RR; 1.709; 95% CI; 0.605–4.831; *p* = 0.312) with statins compared to placebo/control (Fig. 4a-f).

Pooled results from 5 RCTs with 404 patients on statins or placebo/standard treatment demonstrated a non-significant lower risk of elevated CK (RR; 0.891; 95% CI; 0.342–2.319; *p* = 0.813) while the overall pooled results for 490 statin-treated patients versus non-users among non-randomized studies showed an increase in risk of elevated CK (RR; 1.101; 95% CI; 0.457–2.651; *p* = 0.830) but not statistically significant (Fig. 4a-f).

### Risk of bias

About 54% of the cohort studies were of good quality while about 35% were of fair quality (see Supplementary Table S2 online). About 14.3% of the RCTs had a “low” risk of bias, 61.9% “some concerns” risk of bias and 23.8% “high” risk of bias (see Supplementary Fig. S1**a,b** online).

### Meta-regressions and sensitivity analyses

Baseline characteristics (see Supplementary Table S3 online) of HAART-treated PLWH varied widely across included studies (see Supplementary Table S4 online). Also, we combined cohort studies and RCTs in our meta-analysis, but our results indicated low heterogeneity (I^2^ < 50%) for most study outcomes except all-cause mortality which showed high heterogeneity (I^2^ > 70%) with fixed effect meta-analysis. The impact of potential heterogeneity was minimized by using a DerSimonian-Laird random-effect model in analysis. Where applicable, we conducted mixed effect meta-regressions to explore the impact of patients’ baseline characteristics on treatment effects of statins versus placebo/control. The meta-regression models (see Supplementary Table S3 online) confirmed some wide variations in baseline characteristics (see Supplementary Table S4 online). After controlling for study design, year of publication and all other baseline factors, female sex (RR; 1.36; 95%CI; 1.00–1.85; *p* = 0.049), HDL-c (RR; 2.0 × 10^–5^; 95%CI; 3.8 × 10^–10^-1.06; *p* = 0.051) and HIV-RNA copies (RR; 1.00; 95%CI; 1.00–1.01; *p* = 0.047) remained significantly associated with incidence of adverse effects of statin treatment versus placebo/control (see Supplementary Table S3 online). Mixed effect meta-regression did not show any significant association between baseline factors and statin discontinuation rates (see Supplementary Table S4 online).

Sensitivity analyses did not demonstrate any changes in effect estimates for all study outcomes (adverse events, I^2^: 36.98%; discontinuation rate, I^2^: 0.00%; all-cause mortality, I^2^: 77.74%) (see Supplementary Fig. S2-S8 online). In subgroup analyses, rosuvastatin was associated with reduced risk of statin discontinuation compared to placebo/control (RR; 0.633; 95% CI; 0.410–0.979; *p* = 0.040); pravastatin was associated with a non-significant lower risk of statin discontinuation (RR; 0.719; 95% CI; 0.436–1.187; *p* = 0.197). Atorvastatin was associated with higher risk of statin discontinuation but statistically non-significant compared to placebo/control (RR; 2.21; 95% CI; 0.429–11.392; *p* = 0.343). In addition, we found that moderate-intensity dosing was associated with lower risk of discontinuation compared to placebo (RR; 0.684; 95% CI; 0.478–0.978; *p* = 0.037) (see Supplementary Fig. S9-S11 online; Supplementary Fig. S12-S14 online).

### Publication bias

Publication bias was assessed for all outcomes using funnel plots and Begg adjusted-rank correlation^17^ and Egger regression asymmetry tests^18^. We found no significant publication bias for most of the study outcomes except for discontinuation rate.

### GRADE assessment of quality of evidence

The quality of evidence for each of the following outcomes: all-cause mortality, treatment discontinuation, and incidence of adverse effects, gastrointestinal adverse effects, myalgia, diabetes mellitus, elevated CK, and elevated liver enzymes was assessed using the GRADEpro online tool. Certainties of the absolute effects of statins on the assessed outcomes were moderate to high indicating high quality of evidence (See Supplemental Table S5).

## Discussion

We compared the effects of statins on all-cause mortality, discontinuation rates, and incidence of adverse effects with placebo/control in HAART-exposed PLWH using a meta-analysis of 21 RCTs and 26 cohort studies involving 91,594 individuals followed up for a median of 3 months (IQR, 0.8–49.3 months). Compared to placebo/control, statins were associated with significantly lower discontinuation rates. In addition, statin did not lower the risk of myalgia or liver enzyme elevations in PLWH on HAART. Statin did not significantly reduce all-cause mortality or the incidence of adverse events, diabetes mellitus, increased CK elevation, or gastrointestinal side effects in PLWH with prior HAART exposure.

In a recent meta-analysis of 9 cohort studies comprising 73,256 people, Li and colleagues discovered that statins were associated with a 44% reduction in all-cause mortality^42^. Moreover, a meta-analysis of 7 cohort studies in PLWH with/without HAART showed that statins were linked to a 33% reduction in all-cause mortality^43^. The larger sample sizes and the proportion of patients on statins in the studies included in prior meta-analyses may account for the statistically significant reduction in mortality compared with placebo. However, we found a modest reduction in all-cause mortality, which was not statistically significant. This study supports recent findings by Phan and colleagues who demonstrated no significant difference in all-cause mortality between statin users and non-users (HR; 0.74; 95% CI; 0.17–3.29; *p* = 0.70)^5^. According to Lang et al., statins have the same effect on death from all causes as a placebo (HR; 0.86; 95% CI; 0.34–2.19)^6^.

Our meta-analysis on all-cause mortality involved data from 5 cohort studies of PLWH on HAART with follow-up durations from 38–156 months. Statins did not significantly lower all-cause mortality among PLWH in our pooled results, which is consistent with earlier research by Rasmussen et al.^44^, Lang et al.^6^, and Phan et al.^5^. Conversely, our results did not support the findings of Moore et al. and Bedimo et al.^45,46^. The longer follow-up duration and larger placebo groupsize observed in Moore et al.’s study may explain the significant reduction in all-cause mortality seen with statin treatment compared with control/standard treatment. Moreover, Bedimo et al.’s^46^ results do not corroborate our findings plausibly due to their longer follow-up, older HIV-infected patients, and fewer female patients.

The discontinuation rate is an important factor in the assessment of a drug’s effectiveness in routine practice^47^. Our meta-analysis of 13 RCTs (n = 878) and one cohort study (n = 86) revealed that the discontinuation rate among PLWH taking statins was significantly lower than that of non-statin users. This may be due to the fact that the subjects included in our study were younger and that some of them (~ 50%) had never used protease inhibitors. Use of protease inhibitors and advanced age are known risk factors for statin toxicity and consequently statin-related side events. In the studies that were included, self-discontinuation and loss to follow-up were the most common reasons for stopping statin/control medication^8–11,25,48,49^ while statin-related adverse effects such as elevated CK and liver enzymes levels and myopathy were less common reasons^7,8,11,25,33^.

Our study does not support findings of Gili et al.’s meta-analysis of 736 HAART-exposed PLWH^26^ that found a higher incidence of statin discontinuation (MD, 0.12 per 100 person-years; 95% CI 0.05–0.20). Patients had HIV infection for 8.8 years; were exposed to HAART for about 5.4 years and commonly used 10 mg atorvastatin as statin treatment. Patients included in Gili et al.’s meta-analysis had few differences from ours in terms of patients’ characteristics which includes; more female subjects (median, 21.0% vs. 17.0%; IQR: 13.2–25.5% vs. 0–80.8%) and protease inhibitor use (76.5% vs. 50.1%), lower BMI (median 23.9 vs. 25.1; IQR: 23.3–25.1 vs. 22.4–30.0), and 10 mg atorvastatin use (vs. 10 mg rosuvastatin and 20–40 mg pravastatin use). Low BMI, female sex, statin class, and protease inhibitors have been identified as risk factors of statin toxicity^50^. Protease inhibitors block the CYP34A pathway and as a result produce high and toxic plasma levels of lipophilic statins like atorvastatin. Lipophilic statins are more likely to cause myopathy because they more readily permeate into skeletal muscle^50^. This could explain why the risk of statin discontinuation in this study, was lower than with control or placebo group.

In contrast, our data support a prior meta-analysis by Riaz et al. that included 17,770 patients (OR. 0.99; 95% CI 0.93–1.06) and found that patients receiving placebo had higher but non-significant discontinuation rates than those receiving statins (13.9% (n = 8,898) vs. 13.3% (n = 8872))^35^. Our pooled results provide additional support for findings from another meta-analysis of 19 RCTs involving 71,344 patients exposed to statins and those exposed to a placebo, which found no association between statin treatment and a lower risk of discontinuation (RR; 0.95; 95% CI: 0.75–1.21)^51^. Additionally, our study supports the findings of previous RCTs^8,25,48,49^ that showed statin-unexposed PLWH had greater discontinuation rates than statin-exposed PLWH.

Our subgroup analyses revealed that rosuvastatin was significantly associated with lower risk of statin discontinuation relative to placebo (RR; 0.633; 95% CI: 0.410–0.979; *p* = 0.040) in PLWH on HAART. This could be explained by the fact that pravastatin (hydrophilic) is solely metabolized by OAT 1, whereas rosuvastatin (hydrophilic) is a substrate to both organic anion transporting polypeptides 1 (OAT 1) and cytochrome P450^52^. Compared to lipophilic, hydrophilic statins are less likely to cause myopathy because they less readily permeate into skeletal muscles. Moreover, protease inhibitors are able to produce toxic plasma levels of lipophilic statins like atorvastatin, which result in higher rate of statin discontinuation^50^. Our subgroup analysis focusing on the impact of statin intensity demonstrated a 31.6% lower risk of discontinuation with moderate-dose statin versus placebo (RR; 0.684; 95% CI: 0.478–0.978; *p* = 0.037). On the contrary, Gili et al. found higher risk of statin discontinuation with moderate-dose rosuvastatin (MD; 0.44; 95% CI: 0.22–0.65; *p* < 0.0001)^26^. Additionally, our research does not corroborate the findings of Vinogradova et al.’s study, which revealed that statin type and dose are not important determinants of discontinuation^53^. Gili and colleagues defined statin discontinuation as cessation of treatment based on statin-related adverse effects. In contrast, we took into account side effects related to statins, self-withdrawal, and loss to follow-up.

Statin-related adverse events in PLWH on HAART are a major reason for statin discontinuation. The high prevalence of myalgia among PLWH may be attributed to statin class, statin dose, protease inhibitors, concurrent use of medications such as fibrates and niacin, advanced age (> 75 years), female sex, low BMI, alcoholism, and vitamin D deficiency. We found no significant association between statin treatment and increased risk of myalgia and elevated levels of liver enzymes and CK in PLWH. Also, no significant association of statins with risk of any adverse effects, gastrointestinal symptoms, and diabetes mellitus was found. Our findings are consistent with results from Calza et al.’s study, which found that myalgia and increased CK activity are prevalent in HAART-treated PLWH on statins but are not significantly associated with statins^7^. Similarly, a meta-analysis of eighteen studies (n = 106,321) found equal rates of myopathy between statins and placebo groups of PLWH (OR; 1.2; 95% CI = 0.88–1.62)^35^. However, a recent meta-analysis by Cai et al. found a significantly elevated incidence of myalgia (21 RCTs, n = 65, 304) and liver dysfunction (21 trials, n = 54,803) among the HIV-uninfected persons with or without statins^54^. This disparity could be explained by the fact that majority of Cai et al.’s pooled studies that indicated liver dysfunction included patients on 10–80 mg atorvastatin. Statin-induced hepatotoxicity can be caused by: i) inhibition of the mevalonate pathway; ii) changes in lipid composition of the hepatocyte membranes; and iii) lipophilicity with high oral daily dose of statin^50^.

Statins, particularly high-dose statin and rosuvastatin, increases the risk of developing new-onset diabetes mellitus^50^. Atorvastatin lowers the concentrations of glucose transporter 4 and caveolin-1 in skeletal muscle^50,52^. The SATURN (Stopping Atherosclerosis and Treating Unhealthy bone with Rosuvastasin in HIV) trial and HIV Outpatient (HOP) cohort study both found significant associations between of statin use and diabetes mellitus^55,56^. In contrast, a cohort study of 6,195 patients conducted by Spagnuolo et al. complements our findings by demonstrating that statin use was associated with a non statistically significant increase in incidence of diabetes mellitus (HR: 1.21; 95% CI: 0.71–2.07; p = 0.47)^57^. Only two trials^9,11^ of the 47 included studies reported incidence of diabetes mellitus among HIV patients on statin treatment.

## Strength and limitations

To the best of our knowledge, we are the first meta-analysis to investigate the effects of statin therapy on rates of discontinuation and adverse events among PLWH. Using the Newcastle–Ottawa scale, all the studies included in our meta-analysis on all-cause mortality were of good quality.

Our meta-analysis was without limitations. First, no identified RCTs reported all-cause mortality, and our meta-analysis comprised few cohort studies. Second, cardiovascular mortality was not pooled since only one trial reported it. Thirdly, dosage, type, and follow-up varied considerably among the included RCTs. However, our meta-regressions and subgroup analyses investigated the influence of sources of heterogeneity on treatment outcomes. Finally, only English-language data were included, resulting in the likely omission of trials published in other languages. However, our assessment of publication bias did not reveal significant bias for the majority of the study outcomes.

## Conclusions

The discontinuation rate of statin-treated PLWH is considerably lower than placebo/control patients. Among PLWH, statin treatment did not significantly lower mortality from all causes. Clinicians should consider the nocebo effect when treating PLWH who are taking statins and have common adverse effects such as myalgia and, elevated levels of CK and liver enzymes.

## Supplementary Information

Supplementary Information.

## Data Availability

Data can be obtained from the corresponding author on reasonable scientific request. Requests to access the datasets should be directed to Njeodo Vigny, N.Vigny64@gmail.com.
