# Supplementary material for: Effectiveness and safety of statins on outcomes in patients with HIV infection: a systematic review and meta-analysis
Source: Sci Rep. 2022 Oct 27;12:18121. doi: 10.1038/s41598-022-23102-2 (PMC9613890; doi:10.1038/s41598-022-23102-2)
Supplement: Supplementary file 1 — Supplementary Information. [file 41598_2022_23102_MOESM1_ESM.docx]

**Supplementary Table S1.** HIV infection features of enrolled patients

|  | Median | Interquartile range |
| --- | --- | --- |
| Baseline CD4+ T-Cell Count (X10^6^ cells/L) | 493 | 85 – 682 |
| HIV-RNA, copies/mL | 50 | 13 – 36186 |
| Time from HIV diagnosis (years) | 11.5 | 0.98 – 16.0 |
| Time on ART (months) | 45.0 | 3.0 – 147.0 |
| PI-containing regimen (%) | 50.1 | 17.1 – 66.7 |

**Supplementary Table S2.** Quality assessment of cohort studies

| **Author's name** | **Study design** | **Representativeness of the exposed cohort** | **Selection of non-exposed cohort** | **Ascertainment of exposure** | **Demonstration that outcome was not present at start of the study** | **Comparability** | **Assessment**  **of outcome** | **Was follow-up long enough for outcome to occur** | **Adequacy of follow-up of cohorts** | **Total** | **Grade** |
| --- | --- | --- | --- | --- | --- | --- | --- | --- | --- | --- | --- |
| Visnegarwala et al | Retrospective cohort | 1 | 0 | 1 | 0 | 0 | 1 | 1 | 1 | 5 | Fair |
| Benesic et al | Prospective cohort | 0 | 0 | 0 | 1 | 0 | 0 | 1 | 1 | 3 | Poor |
| Johns et al | Retrospective cohort | 1 | 0 | 0 | 0 | 0 | 0 | 1 | 1 | 3 | Poor |
| Townsend et al | Retrospective cohort | 1 | 0 | 1 | 1 | 0 | 1 | 1 | 0 | 5 | Fair |
| Rahman et al | Retrospective cohort | 1 | 0 | 1 | 1 | 0 | 1 | 1 | 1 | 6 | Fair |
| Silverberg et al | Retrospective cohort | 1 | 0 | 1 | 1 | 2 | 1 | 1 | 0 | 7 | Good |
| Singh et al | Retrospective cohort | 1 | 0 | 1 | 0 | 2 | 1 | 1 | 1 | 7 | Good |
| Moore et al | Retrospective cohort | 1 | 1 | 1 | 1 | 2 | 0 | 1 | 1 | 8 | Good |
| Calza et al | Retrospective cohort | 0 | 0 | 0 | 1 | 2 | 0 | 1 | 1 | 5 | Fair |
| Overton et al | Prospective cohort | 1 | 0 | 1 | 1 | 2 | 0 | 1 | 1 | 7 | Good |
| Rasmussen et al | Prospective cohort | 1 | 1 | 1 | 1 | 2 | 1 | 1 | 1 | 9 | Good |
| Calza et al | Prospective cohort | 1 | 0 | 1 | 1 | 0 | 0 | 1 | 1 | 5 | Fair |
| Calza et al | Prospective cohort | 1 | 1 | 1 | 1 | 1 | 0 | 1 | 1 | 7 | Good |
| Krsak et al | Retrospective cohort | 1 | 0 | 1 | 1 | 2 | 1 | 1 | 0 | 7 | Good |
| Lang et al | Prospective cohort | 1 | 1 | 1 | 1 | 2 | 0 | 1 | 0 | 7 | Good |
| De Socio et al | Prospective cohort | 1 | 0 | 1 | 0 | 2 | 0 | 1 | 0 | 5 | Fair |
| Clement et al | Retrospective cohort | 1 | 0 | 1 | 1 | 0 | 1 | 1 | 0 | 5 | Fair |
| Boccara et al | Prospective cohort | 1 | 0 | 1 | 0 | 2 | 0 | 1 | 0 | 5 | Fair |
| Ou et al | Retrospective cohort | 1 | 0 | 1 | 1 | 2 | 1 | 1 | 0 | 7 | Good |
| Riestenberg et al | Retrospective cohort | 1 | 0 | 1 | 1 | 2 | 1 | 1 | 0 | 7 | Good |
| deFilippi et al | Prospective cohort | 1 | 0 | 1 | 1 | 2 | 0 | 1 | 1 | 7 | Good |
| Phan et al | Prospective cohort | 1 | 0 | 1 | 1 | 2 | 1 | 1 | 1 | 8 | Good |
| Calza et al | Prospective cohort | 0 | 0 | 1 | 1 | 0 | 0 | 1 | 1 | 4 | Poor |
| Boetigger et al | Retrospective cohort | 1 | 0 | 1 | 1 | 1 | 0 | 1 | 0 | 5 | Fair |
| Brunet et al | Retrospective cohort | 1 | 0 | 1 | 1 | 1 | 1 | 1 | 1 | 7 | Good |
| Bedimo et al | Prospective cohort | 1 | 1 | 1 | 1 | 2 | 1 | 1 | 0 | 8 | Good |

**Supplementary Table S3.** Mixed effect meta-regression analysis showing the association between patient characteristics and incidence of adverse events with statin treatment versus placebo/control

| **Patient Characteristics** | **RR** | **95% CI** | ***p*-value** |
| --- | --- | --- | --- |
| **Age (years)** | 1.19 | 0.98 – 1.46 | 0.081 |
| **BMI (kg/m^2^)** | 1.01 | 0.99 – 1.02 | 0.066 |
| **Sex Female** | 1.36 | 1.00 – 1.85 | **0.049** |
| **Follow up (Months)** | 1.29 | 0.85 – 1.95 | 0.227 |
| **Triglycerides (mmol/L)** | 3.74 | 0.61 – 22.80 | 0.152 |
| **HDL-C (mmol/L)** | 2.0x10^-5^ | 3.8x10^-10^-1.06 | **0.051** |
| **HIV-RNA** **copies/mL** | 1.00 | 1.00 – 1.01 | **0.047** |
| **CD4^+^ T-cell (x10^6^ cells/L)** | 1.00 | 1.00 – 1.01 | 0.229 |

**Supplemental Table S4.** Mixed effect meta-regression analysis showing the association between patient characteristics and treatment discontinuation

| **Patient Characteristics** | **RR** | **95% CI** | ***p*-value** |
| --- | --- | --- | --- |
| **Age (years)** | 1.07 | 0.89 – 1.27 | 0.482 |
| **BMI (kg/m^2^)** | 1.00 | 0.99 – 1.00 | 0.687 |
| **Sex Female** | 0.99 | 0.89 – 1.12 | 0.988 |
| **Follow up (Months)** | 0.98 | 0.91 – 1.04 | 0.490 |
| **HDL-C (mmol/L)** | 4.15 | 5x10^-5^- 3.3x10^5^ | 0.805 |
| **HIV-RNA** **copies/mL** | 1.00 | 0.99 – 1.01 | 0.738 |
| **CD4^+^ T-cell (x10^6^ cells/L)** | 1.01 | 0.98 – 1.01 | 0.514 |

**Supplemental Table S5.** GRADE assessment of the quality of evidence

**Question:** Should statins compared to placebo/control be used for Patients with HIV Infection with/without HAART?

| **Certainty assessment** | | | | | | | **№ of patients** | | **Effect** | | **Certainty** | **Importance** | |
| --- | --- | --- | --- | --- | --- | --- | --- | --- | --- | --- | --- | --- | --- |
| **№ of studies** | **Study design** | **Risk of bias** | **Inconsistency** | **Indirectness** | **Imprecision** | **Other considerations** | **Statins** | **Placebo/Control** | **Relative (95% CI)** | **Absolute (95% CI)** |  |  |  |
| **All-cause mortality (follow-up: range 38 months to 156 months)** | | | | | | | | | | | | | |
| 5 | observational studies (Cohort) | not serious | not serious | not serious | not serious | all plausible residual confounding would reduce the demonstrated effect | 754/12705 (5.9%) | 1327/32482 (4.1%) | **RR 0.944** (0.561 to 1.588) | **2 fewer per 1,000** (from 18 fewer to 24 more) | ⨁⨁⨁◯ Moderate | | CRITICAL |

**CI:** confidence interval; **RR:** risk ratio

| **Certainty assessment** | | | | | | | **№ of patients** | | **Effect** | | **Certainty** | **Importance** |
| --- | --- | --- | --- | --- | --- | --- | --- | --- | --- | --- | --- | --- |
| **№ of studies** | **Study design** | **Risk of bias** | **Inconsistency** | **Indirectness** | **Imprecision** | **Other considerations** | **Statins** | **Placebo** | **Relative (95% CI)** | **Absolute (95% CI)** |  |  |
| **Discontinuation of statin therapy (follow-up: range 3 months to 24 months)** | | | | | | | | | | | | |
| 13 | randomised trials | serious^a^ | not serious | not serious | not serious | publication bias strongly suspected dose response gradient | 52/427 (12.2%) | 74/451 (16.4%) | **RR 0.691** (0.498 to 0.958) | **51 fewer per 1,000** (from 82 fewer to 7 fewer) | ⨁⨁⨁◯ Moderate | CRITICAL |
| **Discontinuation of statin therapy (follow-up: mean 12 months)** | | | | | | | | | | | | |
| 1 | observational studies (cohort) | not serious | not serious | not serious | serious^c^ | publication bias strongly suspected all plausible residual confounding would reduce the demonstrated effect dose response gradient | 2/40 (5.0%) | 2/46 (4.3%) | **RR 1.150** (0.170 to 7.794) | **7 more per 1,000** (from 36 fewer to 295 more) | ⨁⨁◯◯ Low | CRITICAL |

**CI:** confidence interval; **RR:** risk ratio

#### Explanations

a. Eight studies (RCTs) out of eleven had 'Some concerns' regarding the risk of bias while four studies had a 'high' risk of bias. Nine of the RCTs had 'Some concerns' with the randomization process.

b. Beggs (p = 0.002) and Eggers (p = 0.003) tests of publication bias were significant.

c. Sample size and number of events are small.

d. Beggs (p = 0.002) and Eggers (p = 0.003) tests of publication bias were significant.

| **Certainty assessment** | | | | | | | **№ of patients** | | **Effect** | | **Certainty** | **Importance** |
| --- | --- | --- | --- | --- | --- | --- | --- | --- | --- | --- | --- | --- |
| **№ of studies** | **Study design** | **Risk of bias** | **Inconsistency** | **Indirectness** | **Imprecision** | **Other considerations** | **Statins** | **Placebo/Control** | **Relative (95% CI)** | **Absolute (95% CI)** |  |  |
| **Incidence of adverse events (follow-up: range 3 months to 24 months)** | | | | | | | | | | | | |
| 12 | randomised trials | serious^a^ | not serious | not serious | not serious | dose response gradient | 48/448 (10.7%) | 53/492 (10.8%) | **RR 0.851** (0.608 to 1.190) | **16 fewer per 1,000** (from 42 fewer to 20 more) | ⨁⨁⨁⨁ High | CRITICAL |
| **Incidence of adverse events (follow-up: mean 12 months)** | | | | | | | | | | | | |
| 1 | observational studies (Cohort) | not serious | not serious | not serious | serious^b^ | all plausible residual confounding would reduce the demonstrated effect dose response gradient | 18/40 (45.0%) | 20/46 (43.5%) | **RR 0.265** (0.081 to 0.865) | **320 fewer per 1,000** (from 400 fewer to 59 fewer) | ⨁⨁⨁◯ Moderate | CRITICAL |
| **Incidence of gastrointestinal adverse effects (follow-up: range 3 months to 12 months)** | | | | | | | | | | | | |
| 4 | randomised trials | serious^c^ | not serious | not serious | serious^d^ | all plausible residual confounding would reduce the demonstrated effect dose response gradient | 9/80 (11.3%) | 11/109 (10.1%) | **RR 0.866** (0.493 to 1.398) | **14 fewer per 1,000** (from 51 fewer to 40 more) | ⨁⨁⨁⨁ High | CRITICAL |
| **Incidence of gastrointestinal adverse effects (follow-up: mean 12 months)** | | | | | | | | | | | | |
| 1 | observational studies (Cohort) | not serious | not serious | not serious | serious^e^ | all plausible residual confounding would reduce the demonstrated effect dose response gradient | 4/40 (10.0%) | 5/46 (10.9%) | **RR 0.796** (0.381 to 1.663) | **22 fewer per 1,000** (from 67 fewer to 72 more) | ⨁⨁⨁◯ Moderate | CRITICAL |
| **Incidence of Myalgia (follow-up: range 3 months to 24 months)** | | | | | | | | | | | | |
| 8 | randomised trials | serious^f^ | not serious | not serious | serious^g^ | all plausible residual confounding would reduce the demonstrated effect dose response gradient | 18/368 (4.9%) | 16/378 (4.2%) | **RR 1.253** (0.655 to 2.397) | **11 more per 1,000** (from 15 fewer to 59 more) | ⨁⨁⨁⨁ High | CRITICAL |
| **Incidence of myalgia (follow-up: mean 12 months)** | | | | | | | | | | | | |
| 1 | observational studies (Cohort) | not serious | not serious | not serious | serious^h^ | all plausible residual confounding would reduce the demonstrated effect dose response gradient | 7/40 (17.5%) | 5/46 (10.9%) | **RR 1.610** (0.554 to 4.678) | **66 more per 1,000** (from 48 fewer to 400 more) | ⨁⨁⨁◯ Moderate | CRITICAL |

**CI:** confidence interval; **RR:** risk ratio

#### Explanations

a. Seven out of ten RCTs had 'Some concerns' regarding the risk of bias while three studies had a 'high' risk of bias.

b. Sample size and number of events are small.

c. Two RCTs out of four had 'Some concerns' regarding risk of bias while one RCT had a 'high' risk of bias.

d. Sample size and number of events are small.

e. Sample size and number of events are small.

f. Five RCTs had 'Some concerns' regarding the risk of bias while two RCTs had a 'low' risk of bias.

g. 95% Confidence interval include RR of beyond 1.25.

h. Sample size and number of events are small.

| **Certainty assessment** | | | | | | | **№ of patients** | | **Effect** | | **Certainty** | **Importance** |
| --- | --- | --- | --- | --- | --- | --- | --- | --- | --- | --- | --- | --- |
| **№ of studies** | **Study design** | **Risk of bias** | **Inconsistency** | **Indirectness** | **Imprecision** | **Other considerations** | **Statins** | **Placebo/Control** | **Relative (95% CI)** | **Absolute (95% CI)** |  |  |
| **Incidence of diabetes mellitus (follow-up: mean 12 months)** | | | | | | | | | | | | |
| 2 | randomised trials | not serious | not serious | not serious | serious^a^ | strong association dose response gradient | 0/91 (0.0%) | 3/96 (3.1%) | **RR 0.272** (0.031 to 2.393) | **23 fewer per 1,000** (from 30 fewer to 44 more) | ⨁⨁⨁⨁ High | CRITICAL |
| **Incidence of elevated creatine kinase (follow-up: range 3 months to 23 months)** | | | | | | | | | | | | |
| 5 | randomised trials | serious^b^ | not serious | not serious | serious^c^ | all plausible residual confounding would reduce the demonstrated effect dose response gradient | 8/210 (3.8%) | 9/213 (4.2%) | **RR 0.891** (0.342 to 2.319) | **5 fewer per 1,000** (from 28 fewer to 56 more) | ⨁⨁⨁⨁ High | CRITICAL |
| **Incidence of elevated creatine kinase (follow-up: mean 24 months)** | | | | | | | | | | | | |
| 1 | observational studies (Cohort) | not serious | not serious | not serious | serious^d^ | all plausible residual confounding would reduce the demonstrated effect dose response gradient | 3/40 (7.5%) | 1/46 (2.2%) | **RR 3.450** (0.374 to 31.866) | **53 more per 1,000** (from 14 fewer to 671 more) | ⨁⨁⨁◯ Moderate | CRITICAL |
| **Incidence of elevated liver enzymes (follow-up: range 4 months to 12 months)** | | | | | | | | | | | | |
| 3 | randomised trials | not serious | not serious | not serious | serious^e^ | all plausible residual confounding would reduce the demonstrated effect dose response gradient | 5/50 (10.0%) | 3/55 (5.5%) | **RR 1.709** (0.465 to 6.214) | **39 more per 1,000** (from 29 fewer to 284 more) | ⨁⨁⨁⨁ High | CRITICAL |
| **Incidence of elevated liver enzymes (follow-up: mean 12 months)** | | | | | | | | | | | | |
| 1 | observational studies (Cohort) | not serious | not serious | not serious | serious^f^ | all plausible residual confounding would reduce the demonstrated effect dose response gradient | 3/40 (7.5%) | 2/46 (4.3%) | **RR 1.725** (0.303 to 9.811) | **32 more per 1,000** (from 30 fewer to 383 more) | ⨁⨁⨁◯ Moderate | CRITICAL |

**CI:** confidence interval; **RR:** risk ratio

#### Explanations

a. 95% Confidence interval includes an RR of beyond 1.25. Also, the number of events is very low.

b. Four RCTs out of five had 'Some concerns' regarding the risk of bias while one RCT has a high risk of bias.

c. Number of events is low.

d. Sample size and number of events are small.

e. 95% Confidence interval includes RR beyond 1.25 and the number of events is low.

f. Sample size and number of events are small.


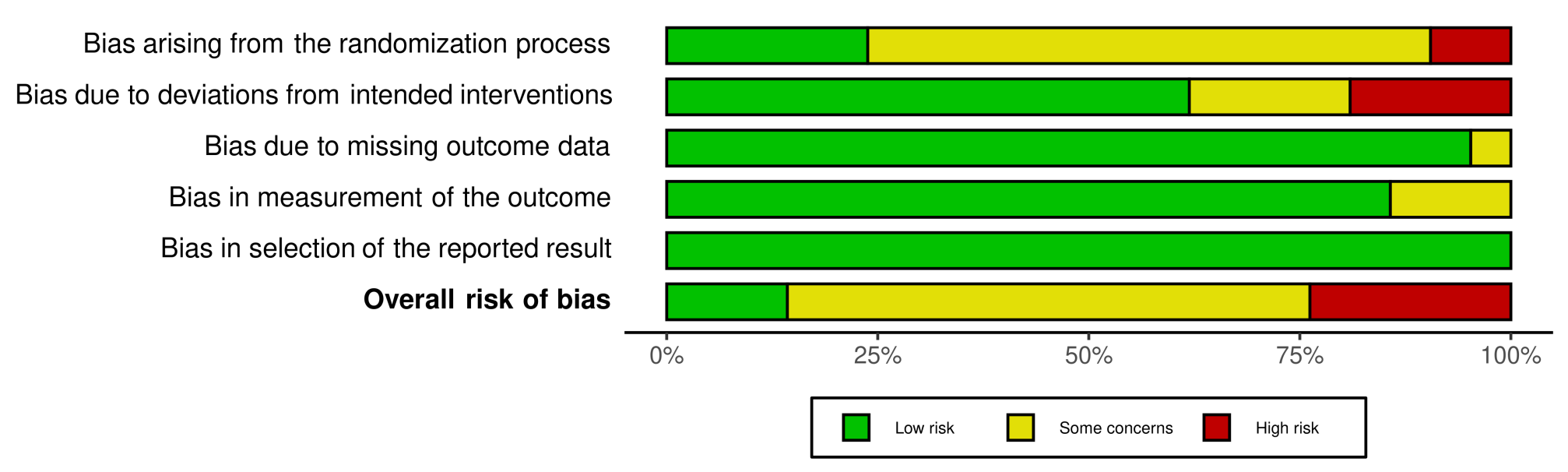


**Supplementary Figure S1, a.** Quality assessment of randomized controlled trials (risk of bias graph)


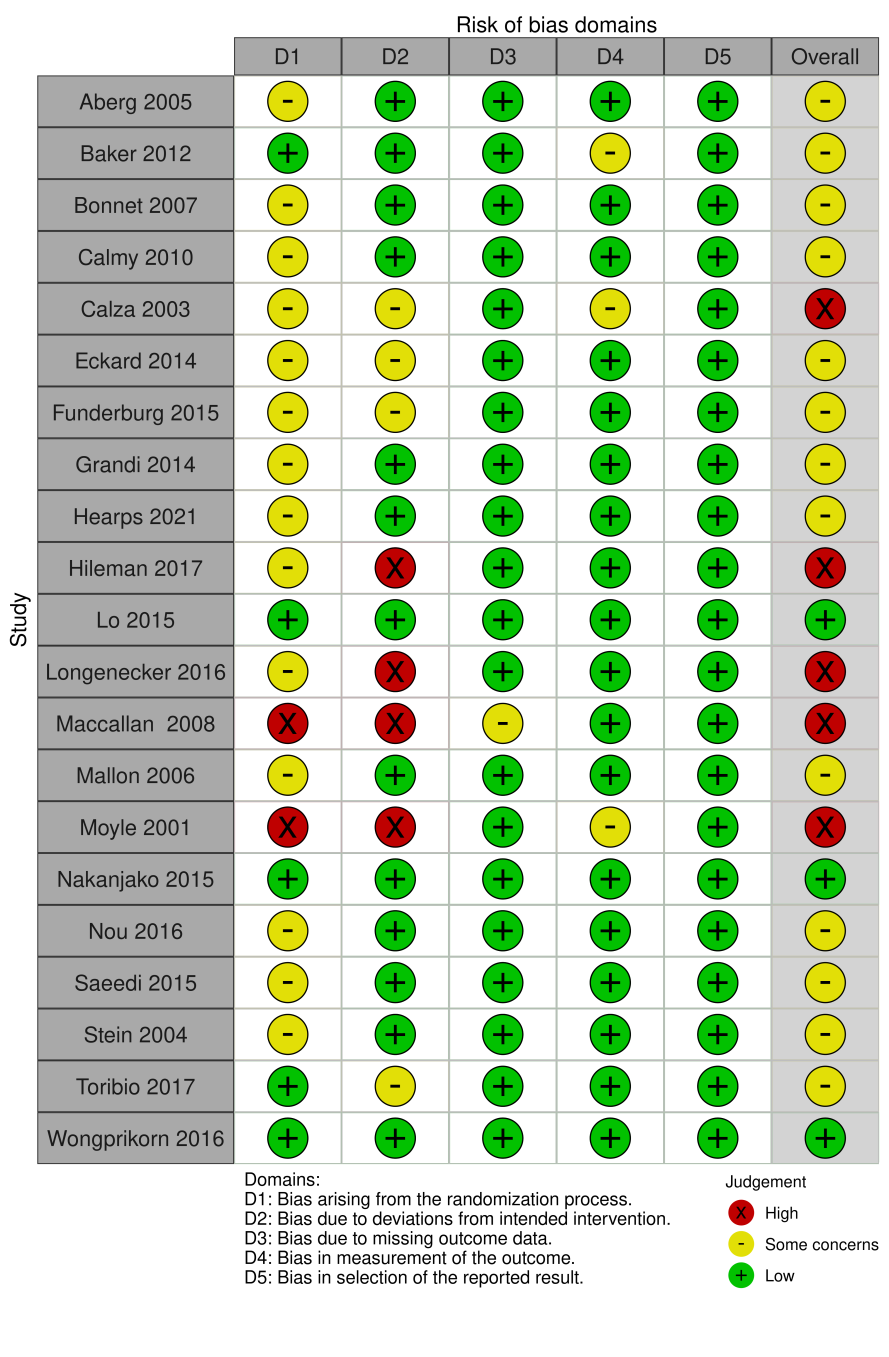


**Supplementary Figure S1, b.** Quality assessment of randomized controlled trials (risk of bias summary)


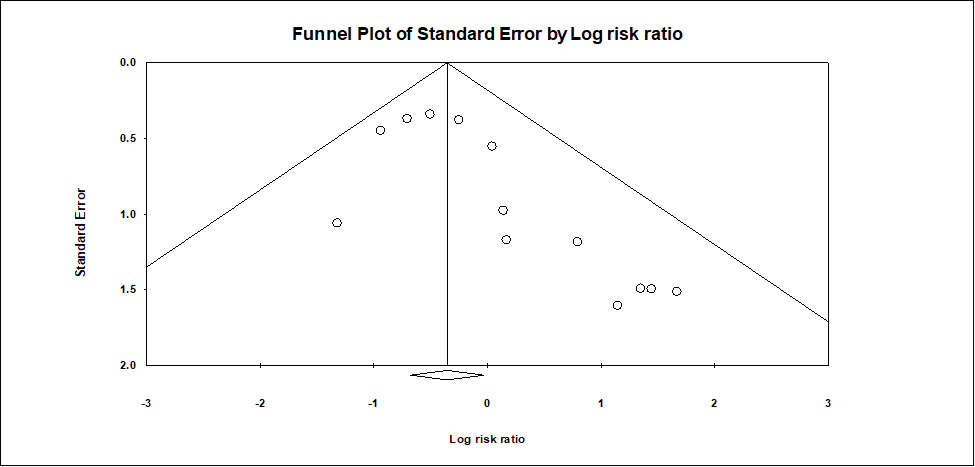


Beggs test (*p* = 0.002); Eggers regression concept test (*p* = 0.003)

**Supplementary Figure S2.** Funnel plot of standard error versus log risk ratio for rate of discontinuation with statin treatment versus placebo/control


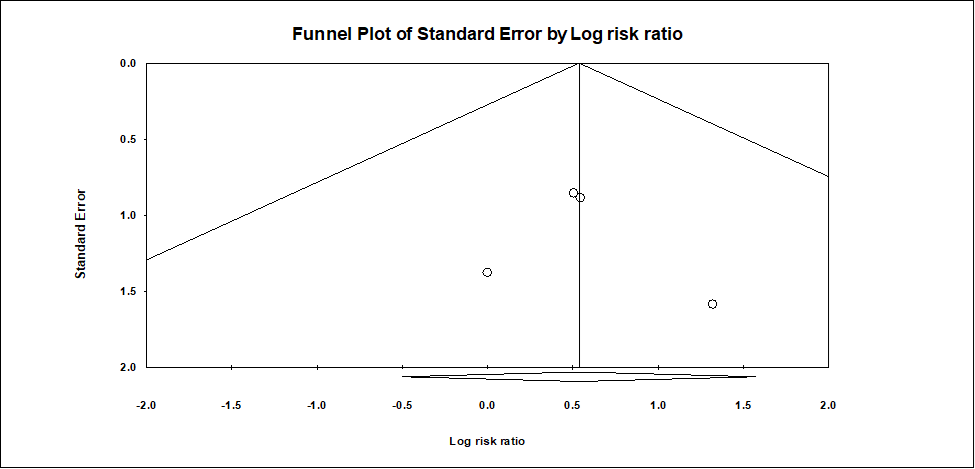


Beggs (*p* = 0.734); Eggers regression intercept test (*p* = 0.745)

**Supplementary Figure S3.** Funnel plot of standard error versus log risk ratio for all-cause mortality with statin treatment versus placebo/control


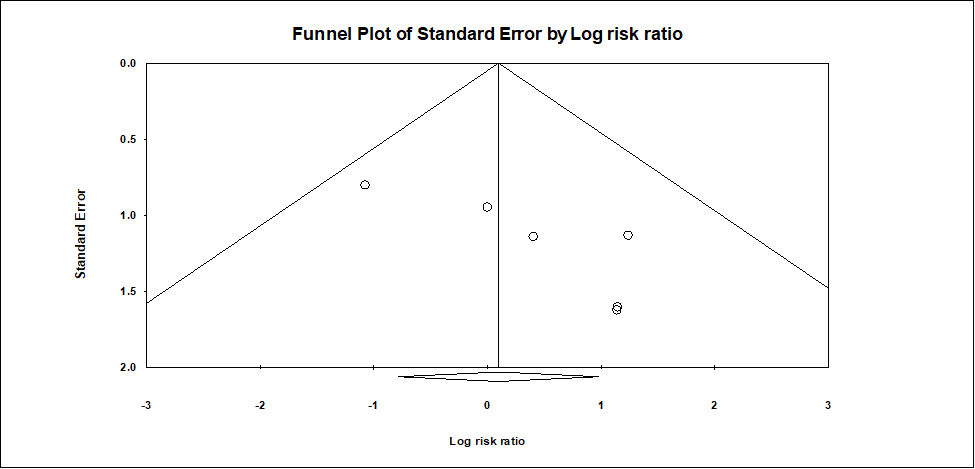


Beggs (*p* = 0.259); Eggers regression intercept test (*p* = **0.039**)

**Supplementary Figure S4.** Funnel plot of standard error versus log risk ratio for CK elevation with statin treatment versus placebo/control


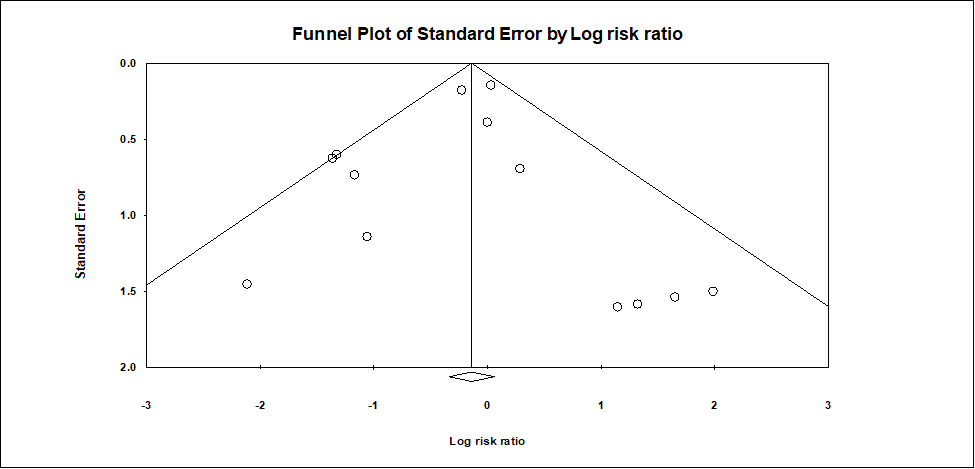


Beggs test (*p* = 0.427); Eggers regression intercept test (*p* = 0.657)

**Supplementary Figure S5.** Funnel plot of standard error versus log risk ratio for incidence of adverse events with statin treatment versus placebo/control


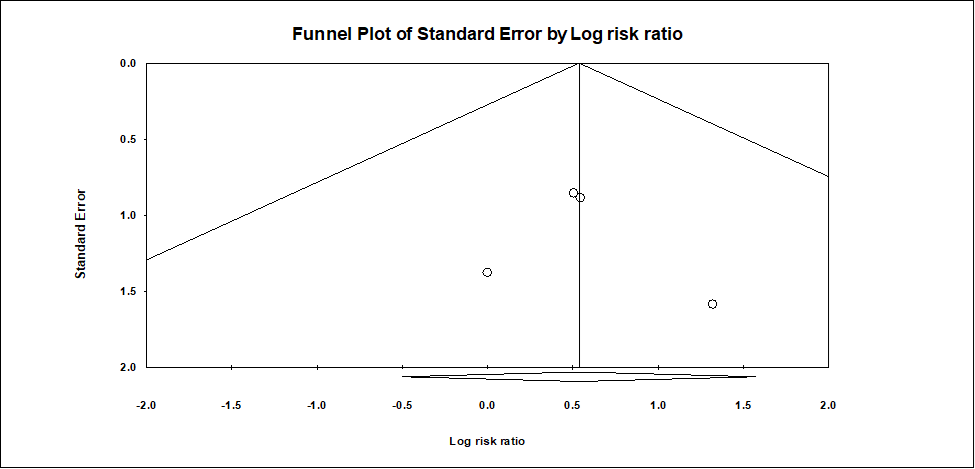


Beggs (*p* = 0.734); Eggers regression intercept test (*p* = 0.745)

**Supplementary Figure S6.** Funnel plot of standard error versus log risk ratio for elevation of liver enzymes with statin treatment versus placebo/control


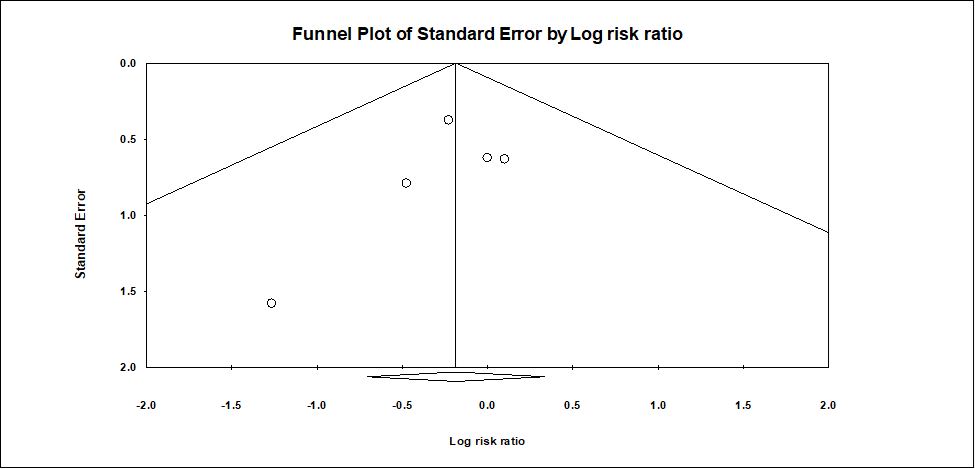


Beggs test (*p* = 0.462); Eggers regression intercept test (*p* = 0.397)

**Supplementary Figure S7.**  Funnel plot of standard error versus log risk ratio for incidence of gastrointestinal (GI) adverse effects with statin treatment versus placebo/control


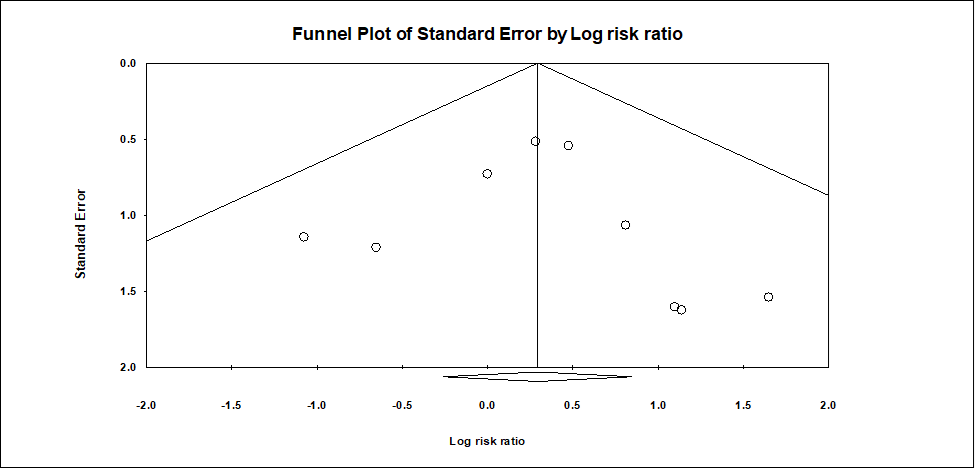


Beggs test (*p* = 0.251); Eggers regression intercept test (*p* = 0.729)

**Supplementary Figure S8.** Funnel plot of standard error versus log risk ratio for incidence of myalgia with statin treatment versus placebo/control

**Supplementary Figure S9.** Forest plot of effect of statin type versus placebo/control on risk of treatment discontinuation in HIV-infected patients on HAART

**Supplementary Figure S10.** Forest plot of effect of statin class versus placebo/control on risk of treatment discontinuation in HIV-infected patients on HAART

**Supplementary Figure S11.** Forest plot of effect of statin dosing intensity versus placebo/control on risk of treatment discontinuation in HIV-infected patients on HAART

**Supplementary Figure S12.** Forest plot of effect of statin type versus placebo/control on risk of adverse effects in HIV-infected patients on HAART

**Supplementary Figure S13.** Forest plot of effect of statin class versus placebo/control on risk of adverse effects in HIV-infected patients on HAART

**Supplementary Figure S14.** Forest plot of effect of statin dosing intensity versus placebo/control on risk of adverse effects in HIV-infected patients on HAART
